# World-First Phase I Clinical Trial for CRISPR-Cas9 PD-1-Edited T-Cells in Advanced Nonsmall Cell Lung Cancer

**DOI:** 10.1055/s-0040-1721451

**Published:** 2020-12-07

**Authors:** Qian Liu

**Affiliations:** 1 Tianjin Lung Cancer Institute, Tianjin Medical University General Hospital, Tianjin, China

Lung cancer is one of the most commonly diagnosed cancers and the leading cause of cancer-related deaths worldwide in 2020. 1 It poses a significant economic burden in developing countries, especially in China. 2 About 80 to 85% of lung cancers are non–small cell lung cancer (NSCLC). 3 The majority of NSCLC patients will be diagnosed with advanced-stage disease. Therefore, the conventional treatment options, including surgery, chemotherapy, and radiotherapy, are unable to result in complete cures. The prognosis of advanced NSCLC remains poor despite years of research into novel combinations of chemotherapy.

Immunotherapy, a new treatment option that uses patient's immune system to fight cancer, holds great promise for cancer patients, 4 including advanced NSCLC patients. 5 6 Immune checkpoint inhibitors, such as antiprogrammed cell death protein 1 (PD-1) humanized antibody, have become the first-line treatment for advanced NSCLC with programmed death-ligand 1 (PD-L1) expression. 7 8 9 The 5-year survival rates of advanced NSCLC patients treated with anti-PD-1 antibodies alone could reach 15.5 to 23.0%. 10 These evidences indicate that blocking the function of PD-1 in T-cells can show a sustained antitumor response in advanced NSCLC patients. However, whether disruption of the PD-1 gene in T-cells of NSCLC patients by gene editing technologies followed by reinfusion of the gene-edited T-cells is therapeutic for advanced NSCLC is still unknown.

CRISPR-Cas9 technology is the most flexible and precise gene editing method currently. 11 12 It edits target DNA by using a guide RNA to help Cas9 recognize the protospacer-adjacent motif upstream or downstream of the target sequence, and then induces Cas9-mediated double-strand breaks in the target DNA. Since CRISPR-Cas9 technology may cause complex genomic alterations and thereby lead to unpredictable consequences, 13 its clinical efficacy is unclear. A recent study published in Science demonstrated the safety and feasibility of CRISPR-engineered T cells in the treatment of refractory cancer. 14 In a study recently published in *Nature Medicine* , titled “Safety and feasibility of CRISPR-edited T cells in patients with refractory non–small-cell lung cancer,” 15 research team led by Prof. You Lu from West China Hospital conducted a world-first in-human phase I clinical trial (NCT02793856) to access the safety and feasibility of CRISPR-Cas9 PD-1-edited T-cell therapy in advanced NSCLC.

In this dose-escalating clinical trial, the authors enrolled 22 advanced NSCLC patients who failed multiple lines of treatment. Among them, 17 (77.3%) patients had sufficient CRISPR-Cas9 PD-1-edited T cells for reinfusion. These patients were divided into four groups (pre-A cohort, cohort A, cohort B, and cohort C). Two patients in pre-A cohort received 2 × 10 ^7^ PD-1-edited T cells per kg body weight and were monitored for 28 days. After confirming that they had no obvious toxicity, the patients in the other three groups received 1 × 10 ^7^ , 2 × 10 ^7^ , or 4 × 10 ^7^ PD-1-edited T cells per kg body weight in a series of three reinfusions on day 1, day 3, and day 5, respectively. The authors monitored the off-target risk by analyzing the edited T cells via next generation sequencing and whole-genome sequencing. Additionally, they tracked the edited T cells through evaluating the peripheral T-cell receptor clone diversity and unique T-cell receptor clones in peripheral blood mononuclear cells.

The authors found that the edited T cells were detectable in peripheral blood during and following the study treatment. All adverse events due to PD-1-edited T-cell therapy experienced by subjects enrolled were grade ½ suggests that the study treatment was well-tolerated. The median progression-free survival and overall survival was 7.7 weeks (95% confidence interval [CI] = 6.9–8.5 weeks) and 42.6 weeks (95% CI = 10.3–74.9 weeks), respectively. The median frequency of off-target mutation events was 0.05%, ranged from 0 to 0.25%.

This study demonstrated for the first time that the clinical application of CRISPR-Cas9 PD-1-edited T-cell therapy is safe and feasible. Additionally, 4 × 10 ^7^ PD-1-edited T cells per kg body weight given in a 28 days cycle is the final dose. In addition to safety and feasibility, another important concern of this study is the incidence of off-target events. Fortunately, the median mutation frequency of off-target cleavage at all sites detected in this study was very low, and most of these mutations were located in introns or intergenic regions, so it is unlikely to have a significant impact on the coding genes. The low incidence of off-target events may be related to the plasmid electroporation strategy, which is adopted in this study, but this needs to be further verified in future studies. Moreover, it is impossible to examine whether the edited T cells can recognize tumor neoantigens due to the small sample size of this study. This should be verified in future studies too. Additionally, the authors do detected the expression of PD-L1 by immunohistochemistry to ensure that the tumor tissues from all enrolled patients were PD-L1 positive, but actually, the positive expression of PD-L1 does not necessarily correlate with PD-L1 dependency. However, this issue was not further discussed in this study. During the primary cell culture and CRISPR-Cas9 editing of T cells, the authors found that some well-transformed T cells failed to proliferate; in contrast, most of the T cells treated by ribonucleoprotein editing obtained enough cell products successfully. This may be related to the poor quality of T cells collected from heavily treated NSCLC patients. Therefore, for patients with a low frequency of tumor-reactive T cells, the therapeutic efficacy may be limited. In future studies, researchers should use superior gene editing approaches to improve the quantities of tumor-reactive T cells.

Overall, the safety and feasibility of CRISPR-Cas9 PD-1-edited T-cell therapy has been demonstrated in a cohort of advanced NSCLC patients. However, considering the limitations of this study, more effective gene-editing technologies should be adopted in future studies.
